# Development and Validation of a Pediatric Endocrine Knowledge Assessment Questionnaire: Impact of ac Pediatric Endocrine Knowledge Assessment Questionnaire Intervention Study

**DOI:** 10.4274/jcrpe.3171

**Published:** 2016-12-01

**Authors:** Nidhi Gupta, Marwan Zidan, Kathleen Moltz, Amita Adhikari, Colleen Buggs-Saxton, Hanaa Zidan, Dania Abushanab, Aida Lteif, Chandra Edwin

**Affiliations:** 1 Mayo Clinic College of Medicine, Division of Pediatric Endocrinology, Rochester, Minnesota, USA; 2 United Arab Emirates University College of Business and Economics, Department of Statistics, Al-Ain, United Arab Emirates; 3 ProMedica Toledo Children’s Hospital, Endocrine and Diabetes Care Center, Toledo, Ohio, USA; 4 Children’s Hospital of Michigan, Department of Pediatric Endocrinology, Detroit, Michigan, USA; 5 Detroit Medical Center, Department of Internal Medicine, Detroit, Michigan, USA

**Keywords:** adolescent, Pediatric Endocrinology, Thyroid, patient education

## Abstract

**Objective:**

While there is general agreement that patient education is essential for compliance, no objective tools exist to assess knowledge in children and parents of children with endocrine disorders. We aimed to design and validate a Pediatric Endocrine Knowledge Assessment Questionnaire (PEKAQ) for congenital hypothyroidism, Hashimoto’s thyroiditis, isolated growth hormone deficiency, Graves’ disease, and congenital adrenal hyperplasia. We evaluated baseline knowledge of children and parents of children with these disorders and assessed impact of educational intervention.

**Methods:**

At baseline, 77 children (12-18 years) and 162 parents of children 1-18 years participated in this prospective intervention study. Educational handouts for five targeted disorders were designed. Following one-on-one educational intervention, 55 children and 123 parents participated. Baseline and post-intervention knowledge scores were compared using McNemar’s test.

**Results:**

Adequate multi-rater Kappa measure of agreement was achieved for children’s (0.70) and parent’s (0.75) PEKAQs. Flesch Reading Ease Score for both PEKAQs (15 questions each) was 65. Post-intervention, significantly higher proportion of parents and children answered majority of questions correctly (p<0.05). Sixteen percent more parents and 22% more children knew their diagnosis correctly (p<0.05). Significant improvement was noted among all participants regarding reason for treatment, steps to take in a situation of missed dose, exercise and diet with these disorders, and long-term prognosis. Parent’s knowledge score was an independent predictor of child’s score.

**Conclusions:**

To our knowledge, this is the first validated PEKAQ that can be used widely in pediatric endocrinology clinics. We noted significant improvement in knowledge of children and parents of children with endocrine disorders.

**WHAT IS ALREADY KNOWN ON THIS TOPIC?**

Endocrine disorders commonly require adherence to lifelong hormone replacement therapy. Incomplete understanding by patient and/or caregiver of the importance of following prescribed treatment is considered a primary barrier to adherence. Educational programs have been shown to provide knowledge to patients and enable them to take better care of their chronic disorders. However, there are currently no validated questionnaires to assess patient knowledge of pediatric endocrine disorders.

**WHAT THIS STUDY ADDS?**

We developed and validated the first ever known Pediatric Endocrine Knowledge Assessment Questionnaire (PEKAQ) for five most common pediatric endocrine disorders. We designed effective teaching tools for these five disorders. Using PEKAQ and the teaching tools, we made significant improvement in knowledge of children and parents of children with these disorders. We describe an effective education model that might guide development of future programs.

## INTRODUCTION

The most common pediatric endocrine disorders, with the exception of type 1 diabetes mellitus, include congenital hypothyroidism, Hashimoto’s thyroiditis, isolated growth hormone deficiency (GHD), Graves’ disease, and congenital adrenal hyperplasia (CAH) (1,2,3,4,5). Endocrine disorders commonly require lifelong hormone replacement therapy. Adherence to treatment is crucial for improved prognosis in a developing child (6). The goal of achieving treatment adherence in pediatrics is hindered by several barriers including lack of adequate time for physician-patient interaction, form and palatability of medication, complexity of medication schedule and implementing it without interrupting a child’s routine (7). However, incomplete understanding by patient and/or caregiver of the importance of following prescribed treatment is considered a primary barrier by most clinicians and researchers (7,8,9,10,11,12). Not surprisingly, overall treatment adherence rate for the pediatric population is 50% (ranging 11-93%) (13), and may be significantly lower for chronic disorders.

Educational programs have been shown to provide knowledge to patients and enable them to take better care of their chronic disorders (14,15,16). Effectiveness of such programs is best evaluated by assessing knowledge of patients before and after educational intervention. While there are several health-related quality of life surveys for pediatrics (17,18,19,20), there are currently no validated questionnaires to assess patient knowledge of pediatric endocrine disorders, except two questionnaires developed by King et al (21) and Dunn et al (22) for CAH and type 1 diabetes mellitus, respectively, though the latter was not developed specifically for children.

We sought to develop and validate a single Pediatric Endocrine Knowledge Assessment Questionnaire (PEKAQ) for congenital hypothyroidism, Hashimoto’s thyroiditis, isolated GHD, Graves’ disease, and CAH. We also aimed to design effective teaching tools for these disorders. Using PEKAQ, we aimed to analyze improvement in knowledge of patients and identify factors underlying this improvement. If the effectiveness of this model is established, it may give directions for future clinic-based educational intervention programs.

## METHODS

### Designing the Pediatric Endocrine Knowledge Assessment Questionnaire

Preliminary twenty-one multiple-choice questions were developed regarding diagnosis, treatment, self-care, sick-day management, and prognosis of the five targeted endocrine disorders. Questions raised during research personnel’s encounters with patients, online parent forums, and frequently asked questions on websites related to these disorders were included. Quality-of-life related questions were not included. Each question had five answer choices, with one correct answer for each disease. A ‘do not know/unsure’ option was included. However, incorrect and ‘do not know/unsure’ answers are reported together for purpose of analysis. Question number 4 was the only open-ended question: ‘Please write names of medications that you/your child takes for the endocrine disorder’. Respondents were allowed to look up names on their medication bottles.

Two questionnaires, one each for children and parents, were designed. Both questionnaires had the same questions and were designed to be easily comprehensible by children 12-18 years and parents or legal guardians of children 1-18 years. Ambiguous, leading, and hypothetical questions were avoided (23,24). Demographic data including the patients’ age, sex, and disease duration were collected.

### Validating the Pediatric Endocrine Knowledge Assessment Questionnaire

Delphi technique was employed to validate the PEKAQs (25), wherein an expert panel comprising of 20 pediatric and adult endocrinologists at Detroit Medical Center, Michigan was formed. The questionnaires were sent to each panel member through an online survey. Each member was asked to evaluate the degree to which they thought each question reflected the knowledge required by children/caregivers living with these endocrine disorders to understand and effectively manage the disorder. They were then asked to rank on a five-point Likert scale whether they agreed or disagreed that the question should be included and whether they thought the multiple-choice answers were suitable and parallel. If they thought a question should not be included as it was, they were asked to indicate if they thought the item should be included if it was modified. A space was provided for any modifications and/or comments (21).

### Study Design

The study was conducted at Children’s Hospital of Michigan (CHM) Pediatric Speciality Center and Etkin Speciality Center (satellite clinic of CHM). Initially, a list of all children 1-18 years who had a pre-existing diagnosis of one of the five targeted endocrine disorders and who were being followed by a pediatric endocrinologist at either of the two centers was prepared. From this list, all patients coming for their visits to the clinic during the duration of study were invited to participate. Written informed consent was obtained from participating parents and from parents or legal guardians of participating children younger than 18 years. Oral assent was obtained from children aged 12 years and written assents were obtained from children aged 13-17 years.

Exclusion criteria included new consultation visit, patients older than 18 years, transient congenital hypothyroidism, participants unable to stay for the entire duration of the education session, unable to read the PEKAQ (blind, non-English speaking/reading), and presence of other chronic disorders (type 1 diabetes mellitus, Down’s and Turner’ syndrome, celiac disease). The study was approved by the Institutional Review Board of Children’s Hospital of Michigan.

### Administering the Pediatric Endocrine Knowledge Assessment Questionnaire

Baseline PEKAQ was administered during participants’ endocrine clinic visit. PEKAQ was scored, with each correct answer worth 1 point and each incorrect or ‘do not know/unsure’ answer worth zero point. This was immediately followed by the educational session as detailed below. At their follow-up clinic visit in 3-6 months, PEKAQ was again administered to participants by the same research team. On an average, each participant spent 9-12 minutes completing the questionnaire. The questionnaire was completed in presence of research personnel to avoid discussion between parent and child. Once the participants had completed their response, the questionnaire was checked for any missed item which was then requested to be completed.

### Educational Intervention

A face-to-face educational intervention session of about 10-15 minutes was developed for each disorder. Each participating child and his/her parent were educated simultaneously. If both parents were present, one parent was requested to volunteer for participation.

Reader-friendly, attractive, and informative handouts were designed separately for congenital hypothyroidism, Hashimoto’s thyroiditis, isolated GHD, Graves’ disease, and CAH. Each handout contained information regarding diagnosis, pathogenesis, treatment, self-care, sick-day management, and prognosis. Information was obtained from literature review on PubMed and Up-to-date, and the websites of the Pediatric Endocrinology Society, Pediatric Endocrinology Nursing Society, American Academy of Pediatrics, Mayo Clinic, National Institutes of Health, CAH Research Education and Support, and American Thyroid Association.

Members of the research team were trained to impart education in an interactive and consistent pattern. Participants were given ample opportunity to ask questions. Subsequently, these handouts were given to participants as take-home material.

### Statistical Analysis

Data were managed on an Excel spreadsheet. All entries were checked for keyboard error. Descriptive statistics and knowledge assessment scores were computed as arithmetic mean and standard deviation. Comparison was made between the pre- and post-intervention variables using paired t-test for normally distributed continuous variables, Wilcoxon-singed-rank test for skewed continuous variables, and McNemar’s test for categorical variables. Multiple linear regression analysis was applied to test the effect of current age, sex, age at diagnosis, duration of diagnosis, and corresponding parent’s score on child’s knowledge score. A p-value <0.05 was considered statistically significant. The IBM SPSS Statistics for Windows, Version 19.0. Armonk, NY: IBM Corp. was used for data analysis.

## RESULTS

### Readability of Pediatric Endocrine Knowledge Assessment Questionnaires

Readability of the PEKAQs was assessed by Flesch Reading Ease score which is based on the average number of syllables per word and words per sentence (9). The Flesch Reading Ease score for both PEKAQs was 77 (reading grade level of 6 or above). The Flesch Reading Ease score for each of the five educative handouts was 55-64 (reading grade level of 6-7 or above).

Responses from Delphi survey were anonymously collected; data were coded and analyzed. After deleting questions with low agreement between the experts’ panel, 15 questions were retained in both the questionnaires. The multi-rater Kappa measure of agreement was 0.70 for children’s questionnaire and 0.75 for parents’ questionnaire. Kappa measurement of 0.70 or above indicates adequate inter-rater agreement.

### Sample Characteristics

**Figure 1:**
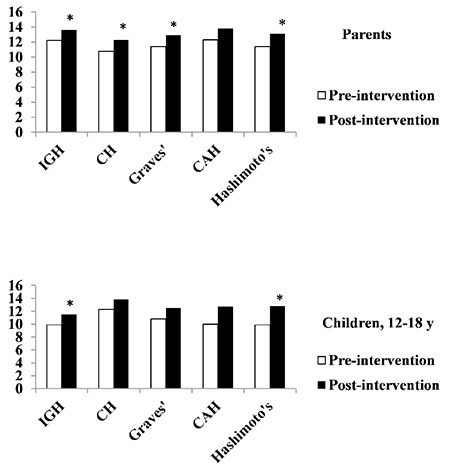
Mean knowledge scores before and after educational intervention by endocrine disorder *p-value <0.05 IGH: isolated growth hormone, CH: congenital hypothyroidism, CAH: congenital adrenal hyperplasia

Of the 162 parents in pre-intervention survey, 79% (n=128) were mothers (Table 1). Post-intervention survey was completed by 123 parents. Among children, 77 participated in pre-intervention survey and 55 completed post-intervention survey. The average age at endocrine diagnosis of participating children was 8.9±4.2 years. Data analysis reported in Tables 2, 3, 4 and Figure 1 includes only those participants who completed pre- as well as post-intervention survey. Reasons for attrition in post-intervention survey included not showing up for follow-up, discontinuation of growth hormone therapy or Grave’s disease treatment, and moving to a different geographical region, thus changing providers.

### Parents: Assessment of Baseline Knowledge

Percentage of parents who answered each PEKAQ question correctly at baseline is given in Table 2. Notably, at baseline, 26.8% parents did not know the correct name of their child’s endocrine disorder and only 63.4% parents knew the reason or beneficial effect of treating their child’s endocrine disorder. Almost 80% parents were not aware of the toxic effects of their child’s endocrine medicine. Less than 70% parents had a sick-day plan and 64.2% knew what should be done in case the child forgets to take his/her medicine at the right time. One-fifth of parents did not know correctly the duration of treatment required, frequency of follow-up at the endocrinology clinic, and reasons for wearing medical alert pendant/bracelet.

### Parents: Impact of Pediatric Endocrine Knowledge Assessment Questionnaire Educational Intervention

Following educational intervention, 10 out of 15 questions were answered correctly by a significantly higher proportion of parents (p<0.05) (Table 2). Specifically, 15.4% more parents correctly knew the endocrine diagnosis of their child (p=0.000) and 36.6% more parents were knowledgeable in recognizing toxic side-effects of their child’s endocrine medicine (p=0.000). Significant improvement in knowledge was observed regarding reason for treatment, steps to take in situations of missed doses, exercise and diet with these disorders, long-term prognosis, and benefit of medical alert pendant/bracelet (p<0.05 for all).

### Children: Assessment of Baseline Knowledge

Table 3 shows the percentage of children who answered PEKAQ questions correctly at baseline. During the pre-intervention survey, 69.1% children knew correctly their own endocrine diagnosis and only 25.5% knew toxic effects of their medicines. Nearly half of the children did not know what should be done if they forget to take their medicine at the right time. About 65% children knew that with their endocrine diagnosis, they do not have dietary restrictions and should consume a well-balanced diet. One-fourth of the participating children were not aware of long-term prognosis of their disorder and if that will affect their education, job, life span, or fertility.

### Children: Impact of Pediatric Endocrine Knowledge Assessment Questionnaire Educational Intervention

After educational intervention, a significantly higher proportion of children answered 7 questions out of 15 correctly (p<0.05) (Table 3). About 22% more children knew correctly their endocrine diagnosis (p=0.002) and 25% more children knew correctly what they should do if they forget to take their medicine at the scheduled time (p=0.007). Further, 89.1% children post-intervention compared to 65.5% pre-intervention recognized that their underlying endocrine disorder does not require any dietary restrictions (p=0.004).

### Predictors of Knowledge Assessment Scores in Parents and Children

Among parents, during pre- and post-intervention surveys, none of the variables including age, sex, age at diagnosis, and duration of diagnosis were found to be independent predictors of knowledge score (Table 4). However, among children, after adjusting for age, sex, and duration of diagnosis, the corresponding parent’s score emerged as an independent predictor of children’s knowledge score during both pre- and post-intervention surveys. In the pre-intervention survey, age was a positive predictor of knowledge score in children; this was not a statistically significant finding during the post-intervention survey, indicating that the intervention made greater improvement in younger children as compared to older children.

### Impact of Pediatric Endocrine Knowledge Assessment Questionnaire Educational Intervention by Endocrine Diagnosis

Following PEKAQ educational intervention, parents of children with isolated GHD, congenital hypothyroidism, Graves’ disease, and Hashimoto’s thyroiditis showed statistically significant improvement in knowledge of these disorders (p<0.05) (Figure 1). In children, improvement was noted in those with isolated GHD and Hashimoto’s thyroiditis (p<0.05). Sample size of children with congenital hypothyroidism, Graves’ disease, and CAH was too small to allow statistically meaningful analysis.

## DISCUSSION

In this study, we have designed and validated an effective model to assess and educate children and parents of children with congenital hypothyroidism, Hashimoto’s thyroiditis, isolated GHD, Graves’ disease, and CAH. To our knowledge, this is the first validated PEKAQ. We have highlighted marked gaps in knowledge of study participants about their endocrine disorders. Encouraging results were observed in post-intervention survey, with significant improvement in knowledge in the majority of participants.

Studies assessing knowledge of children with endocrine disorders are rare (26,27), with no report that we could find, on impact of educational intervention. The PEKAQs in our study were designed specifically for rapid and reliable knowledge assessment in pediatric endocrine patients during clinic visits. In a randomized controlled trial, Sahlqvist et al (28) reported that shortening a relatively lengthy questionnaire significantly increased the response rate. Rosenfeld and Bakker (9) used a 134-question survey to identify key factors that influence compliance in patients receiving growth hormone therapy. Using a 22-question survey, Smith et al (27) reported that 60% of their patients had limited understanding about their growth hormone treatment. King et al (21) developed a 22-question survey to assess knowledge of families living with CAH. We retained 15 questions in the PEKAQs to minimize item non-response rate while allowing efficient measurement of study parameters.

At the first visit with a pediatric endocrinologist, it is often challenging for families to assimilate news of a lifelong diagnosis (29). Focus of the initial visit is to put patient and parents at ease and help them understand the basics of management. Because we believe this visit might be too overwhelming for them, we enrolled participants at follow-up visits.

Previously, a clear relationship has been reported between knowledge of children treated with growth hormone and their degree of compliance and acceptance of treatment (26,30). In our study, at baseline only two-thirds of the participants knew their diagnosis correctly, partly due to several synonyms used for same disorder (Hashimoto’s thyroiditis, acquired hypothyroidism, chronic lymphocytic thyroiditis, autoimmune thyroiditis) or medical acronyms (CAH) for these disorders. Understanding the reason for treatment was noted in only two-thirds of the participants, which is a significant factor in impeding compliance. The improvement in post-intervention knowledge in our study was likely due to the involvement of dedicated research personnel who spent one-on-one time with participants.

Parents play a critical role in management of pediatric chronic disorders (8,31). In fact, we found parents’ knowledge assessment score to be a significant independent predictor of children’s knowledge score. It is therefore paramount to ensure parental understanding of their child’s disorder, ability to recognize toxic effect of medications, and ensure healthy lifestyle. Through this study, parents were educated about continuing endocrine medications in the event of an acute sickness as well as carrying and administering intramuscular corticosteroid for children with CAH, which can be a life-saving intervention. An important highlight of the educative handouts was to communicate to the parents the need to treat their child like any other child without the endocrine disorder, as much as possible. Information about what to expect when beginning a new treatment was also provided.

As of September 2012, 81% of U.S. adults use the internet and, of those, 72% have looked online for health information in the past year (32). The National Institutes of Health recommends that patient education material be written at or below the 6th grade reading level in order to be most effective and understood (33). However, Barnes and Davies (34) reported a mean reading grade level of 13 for 63 online and paper materials on thyroid nodule evaluation and management. Most of these materials had ‘extensive or serious shortcomings’, which lead to uncalled anxiety and misconceptions (35,36). With limited patient encounter time, there is not enough opportunity for all questions to be addressed. A comprehensive yet succinct education module designed in our study will aptly fill that gap. We selected questions following careful review of ‘frequently asked questions’ on various online forums, common concerns expressed by families during their clinic visits, and queries from their primary care providers.

Commonly, the information about endocrine diagnosis in clinical practice is directed towards parents, partly due to the age of the child at initiation of treatment and also because parents are the primary caregivers. Middle school children are capable of understanding their illness and treatment (37). High school children are more likely to think in terms of impact of their disorder on their daily activities (including diet and physical activity), role of treatment, prognosis, and impact on their future relationships including questions regarding fertility, lifespan, and genetics. Our educational handouts were designed to address each of these concerns. Children are afraid to ask questions or tell a physician if they do not understand something. In the present study, children were encouraged to ask questions, share their concerns, and be more involved in their management. A ‘do not know/unsure’ option was included, because it is preferable for the participants to recognize that they are ‘unsure’ of the answer, than for them to ‘think’ they know the correct answer when they are in fact incorrect.

We did not find duration of diagnosis as a significant factor in predicting knowledge score of participants. This finding was supported by King et al (21) who found no statistical relationship between the length of time since diagnosis of CAH and knowledge score (p=0.591). However, in that study, individuals whose total score was <25 (maximum score 44) were generally family members of children diagnosed with CAH more than 10 years ago. This indicates that education is an ongoing process which should address the evolving needs of the family. No significant difference was found between the scores of mothers as compared to fathers in our study, a finding supported by previous studies (21).

The study had certain limitations. Questions related to quality of life were not included as it was felt that such issues could not be assessed adequately in this type of questionnaire. Socioeconomic status, parental education level, and ethnicity data were not collected. Only English speaking/reading participants could be enrolled. Patient population will be considerably more racially and ethnically diverse over the next several decades (38), and efforts are needed to translate these tools and disseminate health information in multiple languages. Long-term follow-up to evaluate changes in attitudes, practices, and outcomes of our study participants was not conducted as part of this study. Future studies to create educational modules for disorders with multi-hormone involvement are required.

In conclusion, we developed and validated a PEKAQ that will be suitable for use in conjunction with education in pediatric endocrinology clinics. The PEKAQ designed in this study is the first tool for this purpose, to our knowledge. It will be invaluable in assessing parental and patient understanding of their disorder and identifying deficits that can be addressed through education. Significant improvement in knowledge of children and parents of children with chronic endocrine disorders was shown in this study. Our education model might guide development of future programs. It is important to start the education process early in the course of management, include adolescents in discussions, and integrate input of pediatric endocrinologists, pediatricians, family physicians, and nurse educators.

## Acknowledgments

We thank Colette Serpetti, RN for assistance with designing educational handouts and Erin Zacharski for assistance with educational intervention and data collection. They have no conflicts of interest.

## Ethics

Ethics Committee Approval: Institutional Review Board at Childrens Hospital of Michigan, Informed Consent: Written informed consent was obtained from participating parents and from parents or legal guardians of participating children younger than 18 years.

Peer-review: Externally peer-reviewed.

## Figures and Tables

**Table 1:**
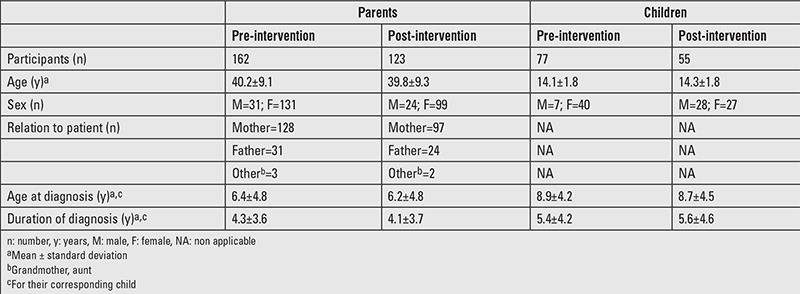
Characteristics of participants

**Table 2:**
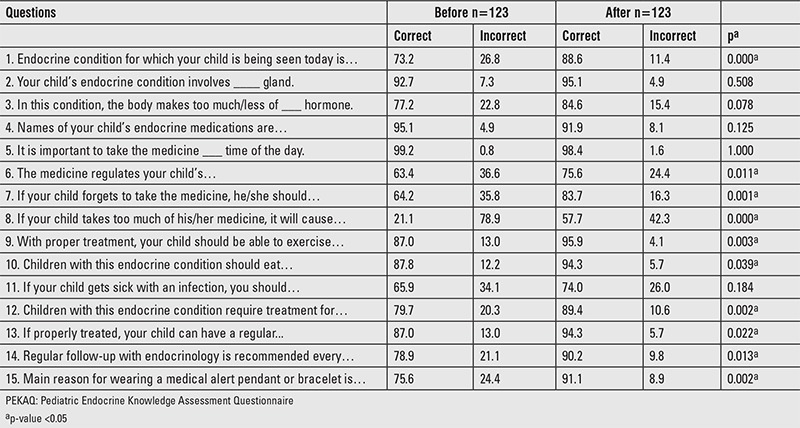
Proportion of parents (%) who answered Pediatric Endocrine Knowledge Assessment Questionnaire questions correctly before and after educational intervention

**Table 3:**
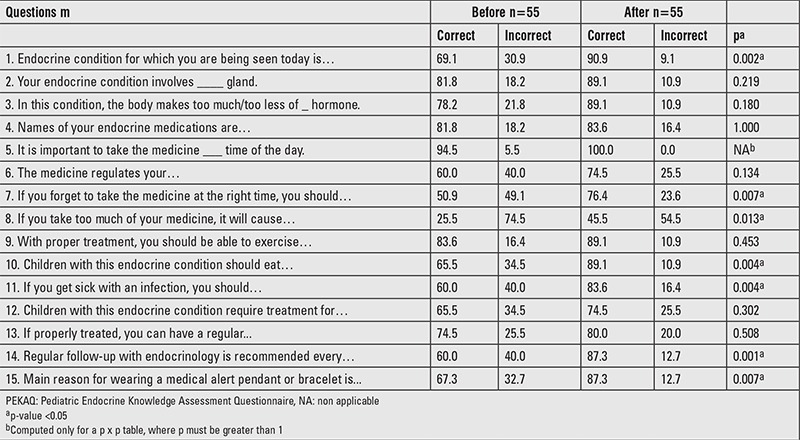
Proportion of children 12-18 y (%) who answered Pediatric Endocrine Knowledge Assessment Questionnaire questions correctly before and after educational intervention

**Table 4:**
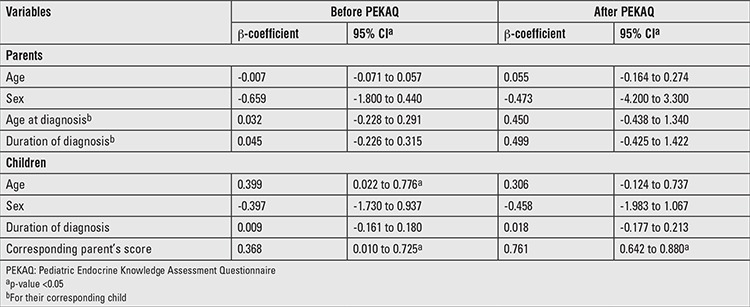
Multiple linear regression for knowledge scores in parents and children
